# A cross-sectional study of self-esteem, psychological factors, and academic performance among occupational therapy students in Japan

**DOI:** 10.20407/fmj.2024-018

**Published:** 2024-12-27

**Authors:** Risa Kayama, Shota Suzumura, Soichiro Koyama, Kazuya Takeda, Kenta Fujimura, Takuma Ii, Hirofumi Ota, Shigeo Tanabe, Hiroaki Sakurai, Yoshikiyo Kanada

**Affiliations:** 1 Graduate School of Health Sciences, Fujita Health University, Toyoake, Aichi, Japan; 2 Faculty of Rehabilitation, School of Health Sciences, Fujita Health University, Toyoake, Aichi, Japan

**Keywords:** Occupational therapy, State-Trait Anxiety Inventory (STAI), Beck Depression Inventory Second Edition (BDI-II), Rosenberg Self-Esteem Scale (RSES), Academic performance

## Abstract

**Objectives:**

With the recent progression of a super-aging society in Japan, demand for medical and welfare professionals has increased, and occupational therapists are in great demand. Academic performance is important for occupational therapy and rehabilitation students. The current study aimed to determine the influence of self-esteem and psychological factors on academic performance in Japanese occupational therapy students.

**Methods:**

A cross-sectional study was conducted among 60 first-year occupational therapy students (16 male and 44 female) at a private medical university in Japan in June 2022. The State-Trait Anxiety Inventory (STAI) and Beck Depression Inventory Second Edition (BDI-II) were used to assess anxiety and depression symptoms, respectively, and the Rosenberg Self-Esteem Scale (RSES) was used to assess self-esteem.

**Results:**

We identified several factors affecting the examination performance of Japanese occupational therapy students. Self-esteem was significantly associated with examination performance in anatomy 1 (p=0.02, β=0.40). RSES-J social work (p=0.07, β=0.31) and anatomy 2 (p=0.08, β=0.30), STAI-JYZ (p=0.09, β=0.38), and STAI-JYZ rehabilitation medicine (p=0.06, β=0.44), and BDI-II (p=0.08, β=–0.43) showed no significant effects but exhibited a trend toward an association.

**Conclusions:**

Understanding the role of psychological aspects and self-esteem is important for constructing systems to support first-year university students. Furthermore, the development of psychological aspects and self-esteem is essential for improving the academic performance of occupational therapy students, and it is crucial to establish educational programs and support systems.

## Introduction

The progression of a super-aging society in Japan in recent years has led to a rapid increase in the demand for medical and welfare professionals in the country.^1^ Among medical professionals, occupational therapists are widely recognized for their ability to support patients to perform specific activities and tasks and thereby improve their quality of life. Occupational therapy students are required to focus on a variety of academic fields and engage in clinical practice and periodic examinations as part of their studies to acquire the specialized knowledge and skills necessary to become occupational therapists.

Academic performance is often considered to be the most important measure of success in education. Educational researchers commonly strive to identify the factors influencing academic performance.^2^ However, students who aspire to become medical professionals face several challenges. First-year students enter specialized fields for the first time, and their academic performance is critical for laying the foundation for their future professional careers. Along with subjects taught in high school, such as physics and biology, knowledge of essential topics for medical professionals, such as anatomy, physiology, and pathophysiology, is also required in the field of occupational therapy. While academic performance at this stage is crucial, student dropout and retention rates tend to be higher in rehabilitation vocational schools, universities, and colleges compared with those in general national and private universities.^3^ The main differences between rehabilitation vocational schools and rehabilitation universities lie in the curriculum and the qualifications awarded. Rehabilitation vocational schools typically offer a 3-year program focused on practical skills and hands-on training. In contrast, rehabilitation universities provide a 4-year academic program culminating in a bachelor’s degree, with a broader curriculum that includes theoretical knowledge, research methods, and general education subjects. University graduates also have the option to pursue advanced studies in graduate school programs. While both types of institutions prepare students for national licensing exams in fields such as physical and occupational therapy, graduate students may have additional opportunities to pursue academic and research careers.

Previous studies have reported several factors associated with academic performance. For example, mental health issues such as anxiety, depression, and sleep problems are commonly observed when students enter university and have been reported to be associated with lower academic performance. For example, a study of general university freshmen indicated that those with internalized mental health problems experienced a detrimental impact on their academic performance.^4^ Furthermore, the 2019 Annual Report by the Center for Collegiate Mental Health highlighted that anxiety was one of the most prevalent mental health issues among general university students who received mental health treatment during the 2018–2019 academic year, with its prevalence continuing to rise.^5^ A study conducted in Japan found that 56% of first-year physical and occupational therapy students exhibited state anxiety and 52% exhibited trait anxiety.^6^ Depressive symptoms have also been shown to significantly impact the academic performance of general university students, leading to an average grade point average decrease of 0.49 points.^7^ Similarly medical and nursing students with high anxiety levels tend to have lower test scores, negatively impacting their academic performance.^8–10^

Self-esteem is also considered to be an essential factor affecting academic performance. A significant correlation exists between self-esteem and academic performance, and higher self-esteem was reported to be associated with better academic performance among rehabilitation university students at the Tehran University of Social Welfare and Rehabilitation,^11^ which is closely related to academic performance through feelings (emotional states) and motivation.^12^ However, to the best of our knowledge, no previous studies have examined the factors influencing academic performance among occupational therapy students in Japan. Identifying the factors that influence student performance, and the strength of their influence is essential for improving academic performance.

Therefore, the current study aimed to determine the influence of self-esteem and psychological factors on the academic performance of Japanese occupational therapy students. Although other medical professions, such as physical therapy and nursing, are equally important, occupational therapists support various aspects of patients’ lives. This role may involve psychological factors and self-esteem issues that are different from other professions, so this study focused on occupational therapy students.

## Methods

### Participants and procedure

The participants in this study were 60 first-year students (16 male and 44 female) majoring in occupational therapy enrolled at Fujita Health University in June 2022. The students were informed of the voluntary nature of their participation in the study. This study investigated the factors related to students’ academic performance upon entering the university. Therefore, occupational therapy students in the second year or higher were excluded from the analysis. Inclusion criteria for this study were as follows: 18 years of age or older, and enrollment in the Faculty of Rehabilitation at Fujita Health University. Exclusion criteria were as follows: severe mental disorders such as bipolar disorder. Students who were repeating a year were excluded from the study because they tended to have strong anxiety symptoms.^13^

Because the current study included human participants, it was reviewed and approved by the Institutional Review Board of Fujita Health University (approval number: HM21-377) and was conducted in accordance with the guidelines of the Declaration of Helsinki. The objectives and procedures of the study were explained to the participants, emphasizing the voluntary nature of participation, anonymity, and confidentiality. Furthermore, the students were informed of the study’s purpose and of the option to withdraw their participation at any time. Written informed consent for participation was not required for this study, in accordance with national legislation and institutional requirements. All participants consented to participation in the study and completed the questionnaires.

### Academic performance

The results of the first semester examinations conducted in 2022 were used to assess the academic performance of the participants. The examinations were conducted between July 14 and September 22, 2022 (including summer vacation). Examinations were conducted in the following areas: five fundamental subjects (biology, statistics, physics, psychology, and communication theory), six specialized subjects (physiology, survey of rehabilitation medicine, social welfare, fundamental information processing, principles of occupational therapy, and principles of physical therapy), and three year-round specialty basic subjects (anatomy 1, anatomy 2, and functional anatomy) (Table 1). In this study, anatomy 1 and 2 and functional anatomy were classified as specialized fields because they contain many specialized elements. Fundamental information processing was excluded from the analysis because it was a report-based assignment.

### Evaluation tool

Previous studies have shown that anxiety, depression, and self-esteem significantly impact academic performance.^7–11^ In this study, we assessed anxiety, depression, and self-esteem. The State-Trait Anxiety Inventory (STAI)^14^ and Beck Depression Inventory Second Edition (BDI-II)^15^ were used to assess anxiety and depression symptoms, respectively. The Rosenberg Self-Esteem Scale (RSES)^16^ was used to assess self-esteem.

The STAI is widely used to assess the intensity of state and trait anxiety. Each item is a two-part scale with 20 items. Both scales are rated on a 4-point scale (1–4) for each of the 20 questions, with total scores ranging from 20 to 80. Higher scores indicate stronger anxiety symptoms. This study used the STAI-Form JYZ (STAI-JYZ), which is sensitive to cultural factors within Japan.^17^ The STAI-JYZ has high internal consistency, and the Cronbach’s alpha coefficient ranged from 0.87 to 0.92 for the state anxiety scale and 0.86 to 0.90 for the trait anxiety scale.^17^ State anxiety reflects a transient response to anxiety-evoking phenomena that change over time. Trait anxiety is the tendency to become anxious, which is derived from a person’s personality and other characteristics. In this study, state anxiety was assessed to determine the current state of anxiety.

The BDI-II is a widely used 21-item self-report scale designed to evaluate depressive symptoms.^15^ Each item is rated on a Likert scale from 0 to 3, with total scores ranging from 0 to 63. The participants in this study rated items on a 4-point Likert scale (ranging from 1=“not at all” to 4=“very much”). This study used an approved Japanese version of the scale, which has been reported to have validity and reliability, with a Cronbach’s alpha value of 0.87.^18^

The RSES is a widely popular self-administered scale comprising 10 items used to assess an individual’s self-esteem. Self-esteem refers to a person’s sense of self-worth or how valuable they feel and how much they approve of, appreciate, respect, value, cherish, and like themselves.^19^ This scale consists of 10 items rated on a 4-point scale ranging from strongly disagree (1) to strongly agree (4). The total score ranges from 10 to 40, with higher scores indicating higher self-esteem. In this study, we used the Japanese version of the RSES (RSES-J), the reliability of which has been confirmed, with a Cronbach’s alpha coefficient of 0.81.^20^

### Statistical analysis

Descriptive statistics were used to calculate the mean (standard deviation) or median (interquartile range) for each fundamental and specialized subject (including specialty basic subjects) and evaluation item. A multiple regression analysis using the forced-entry method was conducted to predict examination performance from the evaluation scales with the total examination score for each subject as the objective variable and the STAI-JYZ, BDI-II, and RSES-J scores as explanatory variables. Regarding multiple regression analysis, there is currently no clear understanding of the permissible extent for using ordinal or nominal scales as explanatory variables. The scores on the STAI-JYZ, BDI-II, and RSES-J, which are ordinal scales, are conventionally treated as ratio or interval scale data.^21^ Therefore, for this study, we treated these values as explanatory variables. The initial model was a constant term alone; the F value for the input of variables criterion was 0.05, and the criterion for exclusion was 0.10. After the multiple regression analyses, the variance inflation factor was calculated to confirm multicollinearity among the explanatory variables. Statistical significance was set at p<0.05. SPSS Statistics Version 26.0 (IBM Corp., Tokyo, Japan) was used for all analyses.

## Results

### Participants’ characteristics

Based on the study’s inclusion criteria, nine participants (one male and eight female) were excluded. The final dataset comprised 51 participants (15 male and 36 female). The results of the examinations (fundamental, specialized subjects including specialty basic subjects) as well as STAI-JYZ, BDI-II, and RSES-J assessments are presented in Table 2. No data were missing.

### Multiple regression analysis with examination score results as the objective variable

Tables 3 and 4 show the results of the multiple regression analysis using the examination scores in each fundamental and specialized subject (including specialty basic subject) area as objective variables. The results showed no significant differences between the examination performance scores for each fundamental subject area and the STAI-JYZ, BDI-II, and RSES-J explanatory variables. In the specialized subject (including specialty basic subject) area, RSES-J (p=0.02) was identified as an independent factor associated with examination performance (anatomy 1). The “standardized partial regression coefficient,” which indicates that the strength of the relative association of the explanatory variable with the objective variable was 0.40. There were no significant differences in the RSES-J of social work (p=0.07, β=0.31), RSES-J of anatomy 2 (p=0.08, β=0.30), STAI-JYZ (p=0.09, β=0.38), STAI-JYZ of survey of rehabilitation medicine (p=0.06 β=0.44), and BDI-II (p=0.08 β=–0.43); however, there was a trend toward association. Interestingly, none of the fundamental subjects showed a significant trend. The variance inflation factor was less than 3 for all variables, indicating no multicollinearity problems.

## Discussion

This study investigated factors influencing academic performance among first-year occupational therapy students. Multiple regression analysis revealed that self-esteem was associated with examination performance in anatomy 1. Furthermore, we found an association between social work (self-esteem), anatomy 2 (anxiety and self-esteem), and introduction to rehabilitation medicine (anxiety and depression), although the differences were not significant. This is the first study in Japan to examine factors affecting the academic performance of occupational therapy students.

In general, anatomy is considered to be essential as a basis for clinical medicine and biology, and is a required field of study for medical and rehabilitation students. The current findings revealed that self-esteem was significantly associated with examination performance in anatomy 1, with a standardized partial regression coefficient of 0.40, indicating a relatively strong association. This result implies that students with higher self-esteem tended to perform better in anatomy. Previous studies have also reported that individuals’ self-esteem, including that of general university students, can predict their academic performance.^22^ In the current study, it is possible that anatomy 1 had different characteristics compared with the other subjects. For example, anatomy 1 focuses on providing detailed knowledge about the structure of the human body and the precise location of organs, which is foundational for understanding more complex anatomical concepts. This foundational knowledge is crucial for further studies in anatomy 2 and functional anatomy. Anatomy 2 builds on this foundation by focusing on the nervous system, including the pathways of neuronal nuclei, neurotransmitter actions, and synaptic information transmission. Functional anatomy further integrates knowledge from anatomy 1 and anatomy 2, emphasizing the functional aspects of anatomy, particularly in relation to movement and rehabilitation. Given these distinctions, the learning content of anatomy 1 covers the basics of human anatomy, which may create a greater sense of accomplishment as students master these essential building blocks. This sense of mastery and achievement may significantly contribute to students’ self-esteem, which might influence their academic performance differently compared with anatomy 2 and functional anatomy. Therefore, the differences in student performance across these courses could be caused by the varying levels of complexity and the level of application required in each.

Although it is common for students to experience anxiety after entering university, they might also experience a range of mental health problems such as depressive symptoms upon entering university.^4^ Prior studies have reported that anxiety and depressive symptoms are associated with lower academic performance.^7–10^ The current results revealed that social work, anatomy 2, and introduction to rehabilitation medicine were related, although the differences were not significant. In particular, the STAI in anatomy 2 and the STAI and BDI in the survey on rehabilitation medicine indicate that students’ psychological characteristics may affect their examination performance. Studies have reported that women tend to experience more anxiety and depressive symptoms than men.^23–25^ In the current study, there were approximately twice as many women (36) as men (15); therefore, it is necessary to consider the influence of sex in experiencing mental health issues.

In the current study, we did not observe significant trends in academic performance related to psychological factors or self-esteem in basic subjects. Basic subjects typically involve foundational knowledge that is less directly tied to the specific skills required for professional practice in occupational therapy. Therefore, students may approach these subjects with a more generalized study strategy, leading to less variation in performance attributable to psychological factors. Furthermore, the assessment methods for basic subjects may differ in format and content from those used in specialized subjects (including specialty basic subjects). Basic subjects might emphasize rote memorization and a fundamental understanding of general principles, while specialized subjects (including specialty basic subjects) often demand higher-order thinking, knowledge application, and the integration of complex concepts related to clinical practice. These differences may play a role in how psychological factors and self-esteem influence academic performance. Students may be more intrinsically motivated and engaged in specialized subjects (including specialty basic subjects) that are directly related to their future profession. This heightened engagement could amplify the impact of psychological factors and self-esteem on their performance in these areas.

The results of the current study suggest that it is crucial to understand the role of psychological characteristics and self-esteem when constructing systems to support students in their first year of university. First-year education must support the critical transition from high school to university. In 2007, the National Institute for Educational Policy Research surveyed national, public, and private universities across Japan and found that first-year education was offered at nearly 97% of all universities nationwide, regardless of whether they were universities, research universities, or colleges.^26^ In first-year education, it may be helpful to consider introducing interventions and support systems to develop students’ self-esteem, particularly in specialty basic subjects such as anatomy. Building a positive self-image and self-confidence may contribute to improved academic performance. Because examination performance in the first year has been reported to be linked to examination performance at graduation,^27^ it is crucial to introduce lectures and curricula to promote self-esteem in the first year of university. Additionally, it is also critical for students to acquire stress-management skills.

A major strength of this study is that it is, to our knowledge, the first to examine the relationship between academic performance, self-esteem, and psychological aspects among occupational therapy students in Japan. The current findings will contribute to the future development of rehabilitation education. However, the study design involved several limitations that should be considered. The design was constrained by a small sample size, which could potentially affect the generalizability of the findings. This issue should have been addressed more carefully during the planning stage of the study. Additionally, the short duration of the study limited our ability to assess long-term effects, which will require more thorough consideration in future research. Furthermore, some potentially relevant factors were not fully investigated, representing a significant gap in the study. For example, self-efficacy,^28^ sleep,^29^ and motivation^30^ should be considered in future studies. These issues reflect deficiencies in the study design and highlight the need for a more robust approach in future research. Additionally, the current study involved several further limitations that should be considered. First, we were unable to compare rehabilitation and general university students. Thus, we were unable to determine whether the predictors of academic performance are unique to rehabilitation students. Therefore, it is necessary to investigate and compare these results with those of general university students. Students training for medical professions often experience a higher level of stress than other students because of the rigor of their professional training. This highlights the distinct psychological and academic pressures that may impact occupational therapy students differently from their peers in more general fields of study. Second, although the current study focused on issues that are specific to occupational therapy students, comparisons with students in other medical fields and further investigation into how occupational therapy students graduate should be conducted. Further discussion may be necessary after conducting a comparative study with students in other medical fields and an analysis of the graduation process for occupational therapy students. Third, we did not conduct a specific analysis of gender differences because of the limited sample size and the exploratory nature of this study. A larger sample size would be necessary to ensure sufficient statistical power to detect meaningful gender differences. Future studies could analyze how gender influences academic performance and psychological well-being among occupational therapy students.

This study used multiple regression analysis to identify the factors affecting the examination performance of Japanese occupational therapy students. Self-esteem was significantly associated with examination performance in anatomy 1. Furthermore, other specialized subjects (including specialty basic subjects) tended to be associated with anxiety and depression among the students. The results also revealed that the development of psychological characteristics and self-esteem is essential for improving the academic performance of occupational therapy students, and it is crucial to establish educational programs and support systems. Further studies that include other factors will be required to draw comprehensive conclusions.

## Figures and Tables

**Table1:** Contents of each subject

Region	Subject	Course overview
Fundamental subjects	Biology	Cell structure, protein structure, cytoskeleton, transport across membranes, signal transduction, energy production, cellular activities such as biological defense, etc.
	Statistics	Descriptive statistics, estimation, tests, etc.
	Physics	Forces and vectors, mechanical energy, laws of motion, etc.
	Psychology	Learning theory, personality, mental mechanisms, developmental stages, interpersonal and group psychology, etc.
	Communication theory	Human cognitive and behavioral mechanisms, communication disorders, etc.
Specialized subjects	Physiology	Brain function, body control, muscle contraction, energy supply for movement, etc.
	Survey of rehabilitation medicine	Knowledge, skills, attitudes, and outline of diseases necessary for rehabilitation medicine, etc.
	Social welfare	Concepts, history, systems, issues in social welfare, and the concept of consultation assistance, etc.
	Fundamental information processing	How computers work, how to use the Internet, how to use document creation software, etc.
	Principles of physical therapy	History, theory of physical therapy, etc.
	Principles of occupational therapy	History, theory of occupational therapy, etc.
Specialty basic subjects	Anatomy 1	Form and function of bones, joints, and muscles, and form and function of organs that comprise the human body, etc.
	Anatomy 2	Action of neurotransmitters, information transmission at synapses, running vasculature in the brain, etc.
	Functional anatomy	Morphology and function of bones, joints and muscles, mainly motor control.

**Table2:** Summary of participant demographics

Variables	Descriptive statistic
Demographic	
Occupational therapy students	51
Age (years)	18.1 (0.3)
Sex	
Men/Women	15/36
Examination results (points)	
1) Fundamental subject	
Biology	64.1 (20.0)
Statistics	75.1 (19.0)
Physics	73.8 (13.2)
Psychology	85.7 (8.8)
Communication theory	86.5 (8.4)
2) Specialized subject	
Physiology	50.5 (22.0)
Survey of rehabilitation medicine	87.5 (8.2)
Principles of physical therapy	67.2 (13.2)
Principles of occupational therapy	85.4 (13.1)
Social welfare	77.0 (10.1)
3) Specialty basic subject	
Anatomy 1	50.8 (23.8)
Anatomy 2	60.3 (17.1)
Functional anatomy	70.4 (19.4)
STAI score (state anxiety)	44.0 (33.0–49.0)
BDI-II score	7.0 (4.0–13.0)
RSES score	26.0 (23.0–27.0)

Data are presented as mean (SD) or median (quartiles). Abbreviations STAI: State-Trait Anxiety Inventory; BDI: Beck Depression Inventory; RSES: Rosenberg Self-Esteem Scale. Differences between Anatomy 1 and Anatomy 2 Anatomy 1: Rehabilitation students learn about the form and function of bones, joints, and muscles as well as the form and function of the organs and tissues that make up the human body. Anatomy 2: Rehabilitation students learn about the pathways of neuronal nuclei and the nervous system, actions of neurotransmitters, information transmission at synapses, and the vascular system running throughout the brain.

**Table3:** Results of multiple regression analysis with examination results [fundamental subjects] as the objective variables

Subject	Independent variables	Standardized coefficients (β)	95% CI		p-value	VIF
			Lower	Upper		
Biology	STAI	0.35	–0.22	1.50	0.14	2.62
	BDI	–0.25	–2.10	0.66	0.30	2.89
	RSES	0.21	–0.66	2.65	0.23	1.50
Statistics	STAI	0.33	–0.23	1.43	0.25	—
	BDI	–0.35	–2.29	0.37	0.25	—
	RSES	–0.01	–1.64	1.55	0.95	—
Physics	STAI	0.09	–0.46	0.69	0.69	—
	BDI	–0.17	–1.26	0.60	0.48	—
	RSES	0.11	–0.77	1.46	0.53	—
Psychology	STAI	0.02	0.92	–0.37	0.41	—
	BDI	–0.10	0.67	–0.76	0.50	—
	RSES	–0.01	0.98	–0.76	0.74	—
Communication theory	STAI	0.31	–0.11	0.61	0.18	—
	BDI	–0.34	–1.00	0.17	0.26	—
	RSES	0.09	–0.51	0.88	0.60	—

Abbreviations: CI, confidence interval; STAI, State-Trait Anxiety Inventory; BDI, Beck Depression Inventory; RSES, Rosenberg’s Self-Esteem Scale; VIF, variance inflation factor. *p<0.05

**Table4:** Results of multiple regression analysis with examination results ([specialized subjects and specialty basic subjects] as the objective variables)

Subject	Independent variables	Standardized coefficients (β)	95% CI		p-value	VIF
			Lower	Upper		
Physiology	STAI	0.22	–0.48	1.41	0.33	2.62
	BDI	–0.04	–1.67	1.39	0.85	2.89
	RSES	0.27	–0.40	3.26	0.12	1.50
Survey of rehabilitation medicine	STAI	0.44	–0.01	0.69	0.06	—
	BDI	–0.43	–1.07	0.06	0.08	—
	RSES	–0.04	–0.75	0.60	0.83	—
Principles of physical therapy	STAI	0.05	–0.51	0.64	0.82	—
	BDI	–0.06	–1.04	0.80	0.79	—
	RSES	0.23	–0.37	1.83	0.19	—
Principles of occupational therapy	STAI	–0.01	–0.63	0.39	0.64	—
	BDI	–0.12	–1.04	0.60	0.59	—
	RSES	0.21	–0.25	2.01	0.20	—
Social welfare	STAI	0.11	–0.33	0.53	0.64	—
	BDI	–0.10	–0.84	0.54	0.66	—
	RSES	0.31	–0.06	1.59	0.07	—
Anatomy 1	STAI	0.16	–0.64	1.36	0.47	—
	BDI	0.06	–1.42	1.80	0.81	—
	RSES	0.40	0.34	4.20	0.02*	—
Anatomy 2	STAI	0.38	0.09	–0.11	0.09	—
	BDI	–0.17	0.48	–1.57	0.75	—
	RSES	0.30	0.08	–0.16	0.08	—
Functional anatomy	STAI	0.13	–0.59	1.05	0.58	—
	BDI	–0.24	–1.99	0.65	0.31	—
	RSES	0.21	–0.60	2.57	0.22	—

Abbreviations: CI, confidence interval; STAI, State-Trait Anxiety Inventory; BDI, Beck Depression Inventory; RSES, Rosenberg’s Self-Esteem Scale; VIF, variance inflation factor. *p<0.05
